# Direct rosiglitazone action on steroidogenesis and proinflammatory factor production in human granulosa-lutein cells

**DOI:** 10.1186/1477-7827-7-147

**Published:** 2009-12-09

**Authors:** Qiuju Chen, Xiaoxi Sun, Junling Chen, Linan Cheng, Jian Wang, Yongwei Wang, Zhaogui Sun

**Affiliations:** 1 NPFPC Key Laboratory of Contraceptives and Devices, Shanghai Institute of Planned Parenthood Research, Shanghai, PR China; 2 Department of Reproductive Medicine, International Peace Maternity and Child Health Hospital, Shanghai JiaoTong University, Shanghai, PR China; 3 Department of Obstetrics and Gynecology, Child and Family Research Institute, University of British Columbia, Vancouver, British Columbia, V6H 3V5, Canada

## Abstract

**Background:**

Ovarian granulosa cells are the predominant source of estradiol and progesterone biosynthesis in vivo. Rosiglitazone, a synthetic agonist of the peroxisome proliferator-activated receptor gamma (PPAR gamma), is applied as the treatment of insulin resistance including women with PCOS. The aim of the study was to investigate the direct effects of rosiglitazone on steroidogenesis and proinflammatory factor production in human granulosa-lutein cells (GLCs).

**Methods:**

Primary human GLCs were separated during in vitro fertilization and cultured in the presence of rosiglitazone, GW9662 (an antagonist of PPAR gamma) and hCG. The mRNA expression of key steroidogenic factors including 3beta- hydroxysteriod dehydrogenase (3beta-HSD), cytochrome P-450 scc (CYP11A1), cytochrome P-450 aromatase (CYP19A1), and steroidogenic acute regulatory protein (StAR) were detected by quantitative real-time PCR. Estradiol and progesterone levels in GLCs cultures were measured by chemiluminescence immunoassay, and the proinflammtory factors (TNFalpha and IL-6) in conditioned culture media were measured by ELISA.

**Results:**

PPAR gamma mRNA levels increased up to 3.24 fold by rosiglitazone at the concentration of 30 microM compared to control (P < 0.05). hCG alone or hCG with rosiglitazone had no significant effects on PPAR gamma mRNA levels. The CYP19A1 mRNA level at exposure to rosiglitazone alone showed a drop, but was not significantly reduced comparing to control. The expression levels of enzymes 3beta-HSD and CYP11A1 in all treatments did not alter significantly. The StAR mRNA expression at exposure to rosiglitazone was significantly increased comparing to control (P < 0.05). The media concentrations of E2 and progesterone by rosiglitazone treatment showed a declining trend comparing to control or cotreatment with hCG, which did not reach significance. Most importantly, treatment with rosiglitazone decreased TNFalpha secretion in a statistically significant manner compared with control (P < 0.05). The concentration of IL-6 following rosiglitazone exposure did not significantly decrease comparing to control.

**Conclusion:**

In cultured GLCs, rosiglitazone stimulated StAR expression, but did not significantly affect steroidogenic enzymes, as well as E2 and progesterone production. Moreover, rosiglitazone significantly decreased the production of TNFalpha in human GLCs, suggesting that PPAR gamma may play a role in the regulation of GLCs functions through inhibiting proinflammatory factors.

## Background

Peroxisome proliferator-activated receptor γ (PPARγ), a well-studied member of the nuclear receptor superfamily, has been implicated in numerous biological processes, including adipocyte differentiation, regulation of lipid and glucose homeostasis, and control of inflammatory responses [1-3]. PPARγ represents important targets for obesity, obesity-induced inflammation, and metabolic syndrome in general. PPARγ agonist, thiazolidinedione (TZD, including troglitazone, rosiglitazone and pigolitazone), is currently used to treat type diabetes (NIDDM) and also to attenuate the secondary clinical symptoms frequently associated with insulin resistance including polycystic ovary syndrome (PCOS) [1-4]. Given that troglitazone has been withdrawn from the market because of hepatotoxicity and one of the important current available PPAR agonists, rosiglitazone, has a binding affinity for PPARγ 100-fold greater than that of troglitazone [3].

In the ovary, PPARγ is expressed most strongly in granulosa cells, and less strongly in theca cells and corpora lutea [5]. The PPARγ is involved in varied processes such as steroidogenesis, angiogenesis, tissue remodeling, cell cycle, apoptosis, and lipid metabolism, which are critical for normal ovarian function. Recently, using the mouse mutant model, granulosa cell-specific deletion of PPARγ resulted in marked impairment of ovulation owing to defective follicular rupture and thereby discovered a critical ovarian function of this factor [5]. Rosiglitazone can directly influence ovarian function and ultimately exert positive effects on oocyte developmental competence in mice [6]. Improved blastocyst quality in obese female mice treated with rosiglitazone before mating indicated that PPARγ was a key target for metabolic regulation of ovarian function and oocyte quality [7].

In recent reports, administration of TZD to women with PCOS resulted in a marked attenuation of hyperinsulinemia that was associated with improvements in insulin secretion, ovarian androgen biosynthesis, and enhanced fibrinolytic system capacity [1,3]. Although it is thought that the inhibitory effects of rosiglitazone on ovarian androgen production are a result of the reduction of circulating insulin levels induced by rosiglitazone, in several *in vitro *and *in vivo *studies in animal and human ovarian cells, PPARγ agonists have been reported to directly inhibit or increase activity of ovarian steroidogenic enzymes, progesterone and estradiol (E_2_) production [8-12]. To clarify the underlying mechanisms, experiments with isolated granulosa cells culture derived from normal women without endocrinological diseases are necessary. Granulose-lutein cells (GLCs) isolated from follicular fluid in *in vitro *fertilization- embryo transfer (IVF-ET) provides us a good source of experiment material.

PPARγ ligands including rosiglitazone also inhibit the activation of inflammatory genes expression and can negatively interfere with pro-inflammatory transcription factor signaling pathways in vascular and inflammatory cells [13]. PPARγ was demonstrated to control a unique network of downstream genes, including those encoding for interleukin-6 (IL-6) and tumor necrosis factor-α (TNFα) [13-15]. Because these cytokines have indispensable effects in ovulation and luteinization [16,17], it is important to understand the regulation of them by PPARγ in the preovulatory granulosa cells, which may be of considerable clinical significance during long-term therapy.

The objectives of this study were to determine the effects of PPARγ agonist rosiglitazone on steroidogenesis and proinflammtory factors production (TNFα and IL-6) in cultured GLCs from infertile women due to tubal blockage.

## Methods

### Subjects

This study was approved by the Institutional Ethics Committee of Shanghai Institute of Planned Parenthood Research. All participants provided their informed consent before inclusion in this study. A total of 20 infertile women with tubal blockage undergoing controlled ovarian hyperstimulation (COH) for *in vitro *fertilization- embryo transfer were enrolled into the study. All of the women had regular menstrual cycles and no evidence of PCOS or other endocrinopathies.

Ovarian stimulation protocols were carried out following our clinical routine described previously [18]. Briefly, Patients in COH cycles received a long stimulation protocol with GnRH agonist and recombinant FSH treatment. The GnRH-a (1.5 mg) was commenced on cycle day 21 of the preceding luteal phase. The starting gonadotropin dose was 150 IU/day, with the dose being adjusted in an individual fashion after 6 days of ovarian stimulation. Ten thousand units of hCG were given *IM *when the leading follicle reached 18 mm and at least three follicles were >15 mm in diameter. Transvaginal ultrasound-guided oocyte retrieval was scheduled 33-36 hours after the hCG injection.

### Follicle fluid collection and GLCs culture

After oocyte retrieval, the follicular-aspirates containing GLCs from 3-4 patients were pooled for GLCs culture. Follicular aspirates were transported on ice to the laboratory and centrifuged at 1000 rpm for 5 min. The pellet was resuspended in PBS (Sigma Chemical Co., St. Louis, MO). The GLCs were separated from the blood cells and cellular debris using 50% percoll gradient centrifugation (Sigma) for 20 minutes. After centrifugation, the interphase cells were collected, washed with PBS by centrifugation for 5 minutes, and incubated with 0.1 mg/mL hyaluronidase (Sigma) for 5-10 minutes in DMEM/F12. The dispersed GLCs were centrifugated, washed and suspended in DMEM/F12 containing 10% FBS, 100 U/ml penicillin, 0.1 mg/ml streptomycin. The GLCs were seeded in a flask. Approximately 1 × 10^6 ^to 10 × 10^6 ^cells were obtained from each patient. Cellular viability was assessed by 0.4% Trypan blue exclusion test and ranged at 70-90%.

After 48 hours, the cells were washed to remove remaining red blood cells which did not adhere to the plastic surface. The cells were then incubated for 24-72 hours at 37°C in DMEM/F12 containing 10% FBS in a humidified atmosphere with 5% CO_2 _in air with following treatments: (1) basic media with 0.01% dimethylsulfoxide (DMSO); (2) different dose of rosiglitazone (7.5 μM, 15 μM, 30 μM, 60 μM) and rosiglitazone 30 μM at different time (24 h, 48 h and 72 h); (3) PPARγ antagonist GW9662 at the concentration of 1 μM; (4) rosiglitazone at the concentration of 30 μM and GW9662 at the concentration of 1 μM; (5) hCG at the concentration of 1 IU/mL; (6) rosiglitazone at the concentration of 30 μM and hCG at the concentration of 1 IU/mL. At the same time, 0.1 μM androstenedione was added to the cultures as substrate for estrogen production. Finally, the conditioned culture media were collected for ELISA and chemiluminent assay. The cultured GLCs were then removed from the dishes by a 5-minute treatment at 37°C with 0.25% trypsin. The cells were collected by centrifugation and stored at -80°C. All the treatments were repeated 6 times.

### RNA extraction, RT reaction and real-time PCR

Total RNA was extracted from GLCs using TRIzol (Invitrogen, Karlsruhe, Germany). Two micrograms of total RNA were reverse transcribed into cDNA. Twenty microliters of RT reaction mixture were used for RT -PCR. Forty nanograms of RNA, 2 μl of RT buffer, 10 IU of RNase Inhibitor, 2 μl of oligo (dT) (10 μM), 2 μl of dNTP-mix (2.5 mM) and diethylpyrocarbonate (DEPC)-treated water up to 10 μl were mixed and incubated at 70°C for 5 min and then immediately placed on ice. Next, 50 IU of Superscript II Reverse Transcriptase was added. cDNA synthesis was carried out at 37°C for 60 min. The reaction was stopped by incubating at 72°C for 10 min. The samples were then stored at -20°C until real-time PCR was performed.

Real-time PCRs were performed using Biorad Chromo4 real-time PCR detector (Bio-rad Co, CA, USA). The reaction mixture consisted of 200 nM of each primer for targeted genes, 12.5 μl SYB green PCR master mix and 9.5 μl of the DEPC water, corresponding to a 0.5 ng mRNA in a total reaction volume of 25 μl. The cDNA was denatured at 95°C for 1 min. The template was then amplified over 36 cycles of 15 s of melting at 95°C, 15 s at 60°C for annealing and 45 s at 70°C for extension. The threshold cycle (Ct) values were normalized to β-actin levels. Relative mRNA levels were calculated using the 2^-ΔΔCt ^method. Primer sequences and the size of the expected PCR products for PPARγ and key steroidogenesis factors including 3β-hydroxysteriod dehydrogenase (3β-HSD), cytochrome *P*-450 scc (CYP11A1), cytochrome *P*-450 aromatase (CYP19A1), and steroidogenic acute regulatory protein (StAR), as well as the endogenous control gene β-actin are presented in Table 1. The specificity of the primers used in the study was confirmed by agarose gel electrophoresis and sequence analysis on the reaction products.

**Table 1:** Primer pairs used for PCR amplification

Gene	**Genebank access no**.	Fragment length (bp)	Forward primer sequence	Reverse primer sequence
PPARγ	NM_005037.5	161	GAGCCCAAGTTTGAGTTTGC	CTGTGAGGACTCAGGGTGGT
3β-HSD	NM_000198.2	196	GCCTGTTGGTGGAAGAGAAG	ATGATACAGGCGGTGTGGAT
CYP11A1	NM_000781.2	200	GGAAATTACTCGGGGGACAT	AACAGACGGAACAGGTCTGG
StAR	NM_000349.2	157	TACGTGGCTACTCAGCATCG	ACAGCAGGCTGGTCTTCAAC
CYP19A1	NM_031226.2	242	CAGAGGCCAAGAGTTTGAGG	ACACTAGCAGGTGGGTTTGG
β-actin	NM_001101.3	271	CTACAATGAGCTGCGTGTGGC	CAGGTCCAGACGCAGGATGGC

### Hormone measurements

Concentrations of E_2 _and progesterone in the culture fluid were determined by chemiluminescence immunoassay using commercial kits obtained from Roche Diagnostics GmbH (Mannheim, Germany). The supernatant was diluted with the Assay Diluent (Roche Diagnostics GmbH) by 50-200 folds when the sample generated value greater than the highest standard. Practical detection limits, within- and between assay coefficients of variation were 18.4 pmol/l, 5.4% and 10.5%, respectively, for E_2 _and 0.095 nmol/l, 6.2% and 9.5%, respectively, for progesterone.

### Measurement of TNFα and IL-6

TNFα and IL-6 concentration in culture media were determined using human ELISA kit (R&D systems, Minneapolis, MN). The lower limits of detectability of TNFα and IL-6 were 12 pg/ml and 10 pg/ml, respectively. All measurements were carried out in duplicate. The intra- and interassay coefficients of variation for TNFα were 8.5% and 10.3%. The intra- and interassay coefficients of variation for IL-6 were less than 7.6% and 10%, respectively.

### Statistical analysis

SPSS 11.5 was used for the data analysis. A one-way ANOVA was used to estimate the statistical difference between the treatment groups. Turkey test was used for variables with normal distribution and the Dunnett's T3 test was used for variables with skewed distribution. A value of *P *< 0.05 was considered significant.

## Results

### Effects of rosiglitazone, GW9662 and hCG on PPARγ expression

**Figure 1:**
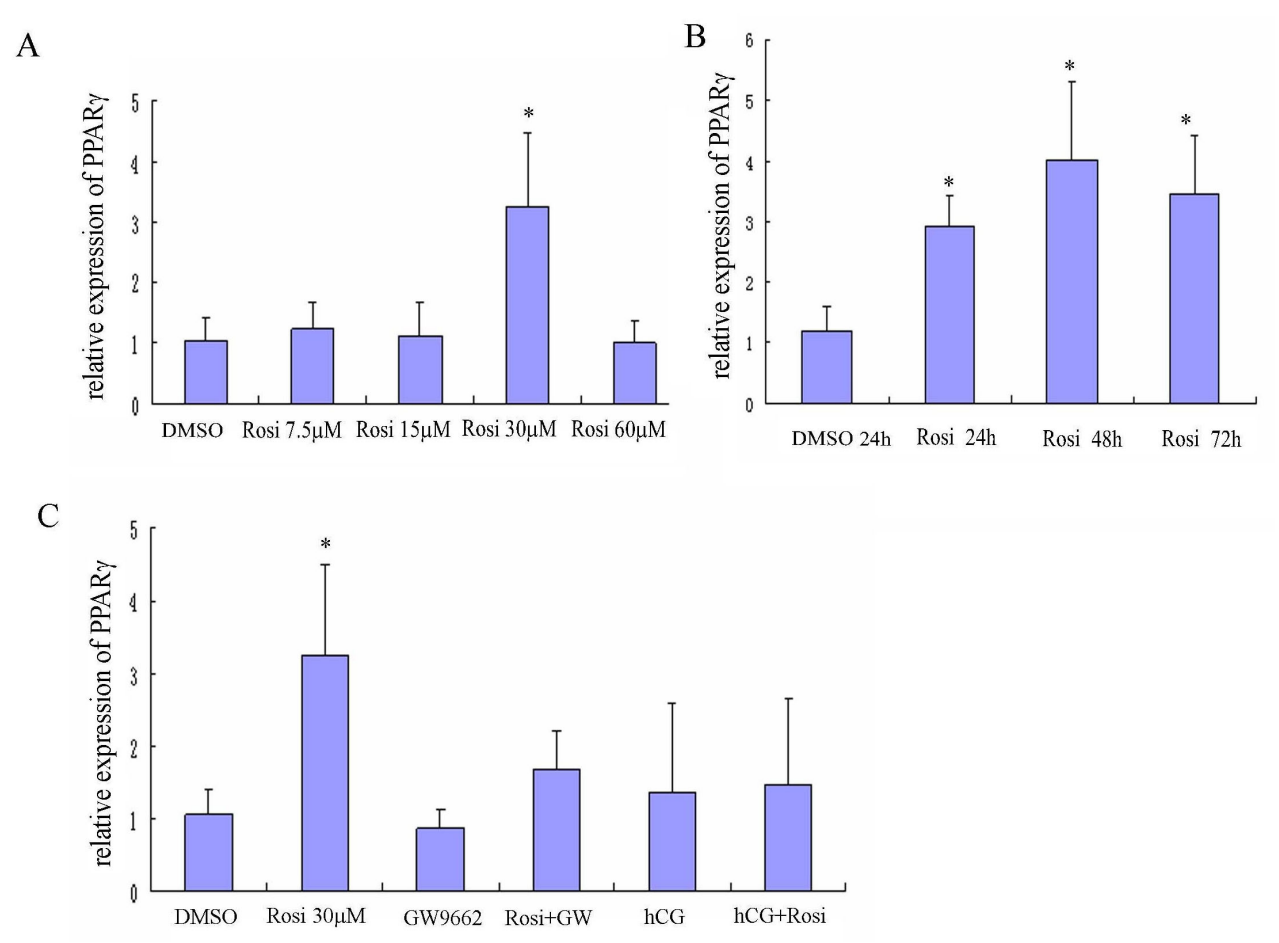
**Effects of rosiglitazone on PPARγ mRNA level in cultured GLCs**. **A**. Relative expression of PPARγ after rosiglitazone (Rosi) treatment for 72 h in different doses. **B**. Time course of rosiglitazone on PPARγ mRNA level in cultured GLCs. **C**. Relative expression of PPARγ in cultured GLCs in response to rosiglitazone treatment for 72 h in the presence or absence of GW9662 and hCG. Results are expressed in ratio to β-actin, and presented as means ± SD of six independent experiments. Statistical significances compared to the DMSO control are shown with one asterisk (*P *< 0.05).

The effect of rosiglitazone in different doses and time course on PPARγ expression in the GLCs is shown in Figure 1. PPARγ expression was stimulated by rosiglitazone which reached a peak point at the dose of 30 μM (3.24 fold compared to a DMSO control, *P *< 0.05). The mRNA expression of PPARγ was increased by rosiglitazone and no difference was found among three time points (24 h, 48 h and 72 h). In the subsequent experiments, the duration of incubation was standardized to 72 h and the dose of rosiglitazone was 30 μM. When the cells were cotreated with GW9662, it partly reversed the effects of rosiglitazone in terms of PPARγ production but no significant difference was achieved. In addition, no significant effects on PPARγ production were shown after treatment with hCG alone or hCG together with rosiglitazone.

### Effects of rosiglitazone, GW9662 and hCG on steroidogenesis

**Figure 2:**
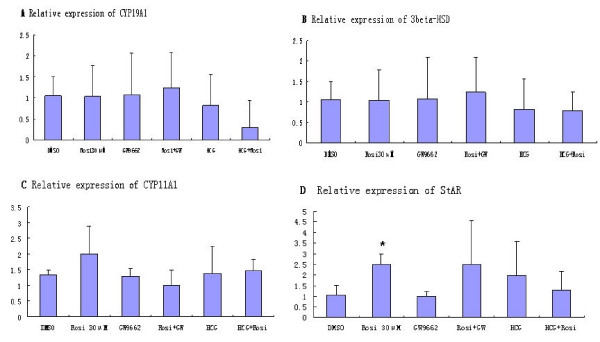
**Regulation of steroidogenic factors by rosiglitazone, GW9662 and hCG in cultured GLCs**. The granulosa cells were treated with rosiglitazone alone, or together with GW9662 and hCG for 72 h. The relative expressions of CYP19A1 (A), 3β-HSD (B), CYP11A1 (C), StAR (D) were measured by real-time RT-PCR. Results in folds of β-actin are expressed as means ± SD of six independent experiments. Statistical significances compared to the DMSO control are shown with one asterisk (*P *< 0.05).

The mRNAs expression of key steroidogenic factors (CYP19A1, CYP11A1, 3β-HSD and StAR) in GLCs are shown in Figure 2. The CYP19A1 mRNA expression following exposure to rosiglitazone showed a reducing trend but no significance was achieved comparing to the DMSO control. Cotreatment with hCG did not change CYP19A1 mRNA levels. The expression of 3β-HSD and CYP11A1 involved in progesterone synthesis did not alter at exposure to rosiglitazone, comparing to control or cotreatment with GW9662 and hCG. On the other hand, the StAR mRNA expression at exposure to rosiglitazone was dramatically increased compared with the DMSO control (*P *< 0.05), and this was attenuated by hCG and GW9662.

**Figure 3:**
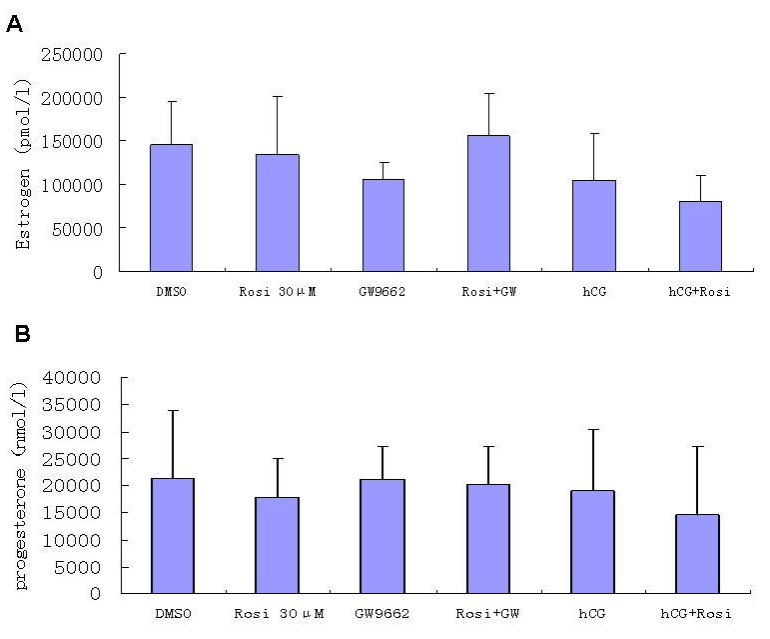
**Effects of rosiglitazone, GW9662 and hCG on steroid hormone production in cultured GLCs**. Concentrations of E_2 _(A) and progesterone (B) in the culture fluid of GLCs treated with or without rosiglitazone, GW9662 and hCG for 72 h. Values are means ± SD of six independent experiments.

The levels of E_2 _and progesterone from the supernatant of GLCs cultures in the presence or absence of rosiglitazone, GW9662 and hCG for 72 hours were also examined (Figure 3). Rosiglitazone alone did not decrease E_2 _and progesterone levels compared with the control. In the presence of GW9662, E_2 _and progesterone levels in the exposure to rosiglitazone also showed no change compared with the control group (*P *> 0.05). Likewise, co-treatment with hCG demonstrated a decreasing trend on E_2 _and progesterone production, but did not achieve a significant difference.

### Effects of rosiglitazone, GW9662 and hCG on TNFα and IL-6 secretion

**Figure 4:**
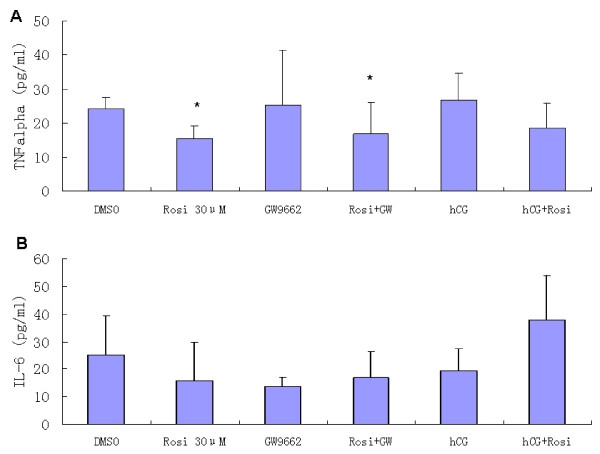
**Effects of rosiglitazone, GW9662 and hCG on TNFα and IL-6 secretion in cultured GLCs**. Concentrations of TNFα (A) and IL-6 (B) in the culture fluid of GLCs treated with or without rosiglitazone, GW9662 and hCG for 72 h. Values are means ± SD of six independent experiments. Asterisk (*) denotes significant differences comparing to the DMSO control (*P *< 0.05).

Secretion of TNFα and IL-6 by cultured GLCs in different treatments is shown in Figure 4. Treatment with rosiglitazone alone or with the presence of GW9662 decreased TNFα secretion in a statistically significant manner compared with a DMSO control (*P *< 0.05). No significant difference was observed with hCG exposure alone, or together with rosiglitazone.

Rosiglitazone decreased IL-6 secretion, whereas did not reach statistical significance. With the presence of GW9662 or hCG, similarly, IL-6 did not change significantly.

## Discussion

In order to explore the possible direct effects of rosiglitazone on human GLCs, we have investigated the production of steroid hormones and proinflammatory factors in GLCs in *in vitro *culture exposure to PPARγ agonist and antagonist. The results indicated that rosiglitazone significantly stimulated StAR mRNA expression, but did not affect steroidogenic enzymes, neither E_2 _nor progesterone production. More interestingly, rosiglitazone significantly decreased TNFα levels in GLCs. Thus, PPARγ may play a role in the regulation of GLCs functions through inhibiting proinflammatory factors.

Despite that *in vitro *studies may not necessarily reflect normal physiology *in vivo, in vitro *experiments may be vital to increase our knowledge of the way in which rosiglitazone influences the function of GLCs. Our data explicated that PPARγ expression was stimulated by rosiglitazone and the greatest response was observed at the concentration of 30 μM. The time course was found no difference among the incubation time 24-72 h. Therefore rosiglitazone 30 μM at 72 h incubation was used for our further experiments and was also approximately comparable to the reports [8,19]. The widely-used selective PPAR antagonist, GW9662, partially reversed the effects of rosiglitazone in terms of PPARγ production, though not significantly. It has been reported that GW9662 could regulate cell growth at high dosages independent of PPARγ [20,21] and this may be the reason for not completely reversing the effect of rosiglitazone.

In nonreproductive tissues, PPARγ has been shown to have a variety of actions within resident macrophage immune cells such as regulating cytokine mRNA expression to modulate overall inflammatory response [15]. TZD activation of PPARγ affect monocyte/macrophage function by downregulating transcription of proinflammatory genes such as inducible nitric oxidase synthase (NOS_2_), IL-1β, IL-6, IL-12 and TNFα [14,15,22,23]. The present study showed for the first time that TNFα was significantly suppressed by rosiglitazone in cultured GLCs. Whereas IL-6 secretion after rosiglitazone treatment appeared to decrease, although this did not achieve significance. TNFα is a multifunctional hormone-like polypeptide and modulates many genes involved in inflammation, infection, and malignancy [17]. Although macrophages are a main source of TNFα, GLCs and corpora lutea have been reported to contain TNFα [24]. TNFα is well known in regulating steroidogenesis, ovulation and luteinization in numerous species including rodents and humans [25-27]. PPARγ played a major role in ovulation because conditional loss of the PPARγ gene in GLCs did not affect ovarian follicular development but prevent follicular rupture [5]. It was supposed that PPARγ may be involved in ovulation and luteinization by inhibiting the secretion of TNFα in GLCs in the present study. The detail mechanism remains unclear and needs further study.

Steroidogenic acute regulatory protein (StAR) plays an essential role in cholesterol transfer from the outer to the inner mitochondrial membrane, thus providing the substrate for steroid hormone biosynthesis [9-11]. Cholesterol is then converted to pregnenolone by the CY11A1 enzyme, initiating steroid biosynthesis. In the present study, rosiglitazone stimulated the StAR mRNA up to 2.6 fold, the results suggested that activation of steroid hormone synthesis by rosiglitazone may be mediated via activation of StAR. In the previous report, the insulin or TZDs up-regulated PPARγ, insulin receptor, insulin receptor substrate-1 and StAR protein expression, so it were presumed that PPARγ, StAR protein and insulin receptor constituted a novel system of ovarian regulation [19].

The steroid hormone production is a multi-step process, requiring action of multiple enzymes. In the present study, the changes of key steroidogenic enzymes (CYP19A1, CYP11A1 and 3β-HSD) induced by rosiglitazone were not obvious in the GLCs. Moreover, the non-significant results for rosiglitazone affecting concentrations of E_2 _and progesterone were coincident with the aboved changes of steroidogenic enzymes. So in the present study, although PPARγ stimulated StAR mRNA expression, the activation of PPARγ did not play a major role in the regulation of steriod hormone production in the cultured GLCs.

In the present study, the effects of rosiglitazone on steroidogenesis were different from some previous reports. Rosiglitazone directly stimulated progesterone and IGFBP-1 production, while inhibited E_2 _and testosterone production using a culture system of mixed ovarian cells [8]. Troglitazone suppressed progesterone production specially by inhibiting the activity of 3β-HSD in porcine granulosa cells [9,11]. In our study, the GLCs isolated from follicular fluid in IVF-ET was in the final stage of differentiation, the responses to rosiglitazone were possibly different from the cells obtained from oophorectomy specimens in human and porcine.

In contrast with the previous report that hCG stimulation induced a transcriptional burst of genes involved in progesterone synthesis (3β-HSD, CYP11A1 and StAR) using luteinized granulosa cells [28], no significant changes were found regarding key steroidogenic enzymes and proinflammatory factors when adding hCG to the GLCs. The fact could not be ignored that the GLCs obtained from IVF patients at the time of oocyte retrieval have received superphysiological doses of gonadotrophins before they were put into culture. The GLCs may have gonadotrophin desensitization due to their over exposition to FSH and LH treatment in vivo so they can not obtain marked response by adding another gonadotrophin stimulant. Similar results were reported in the previous studies [29-31].

In order to determine the action of PPARγ on steroidogenesis in GLCs, more convincing data could be obtained by knocking down of PPARγ expression using human PPARγ siRNA in cultured GLCs. Given that primary GLCs are difficult to transfer using siRNA, the immortalized granulosa cell lines will be good candidate for studying the regulation mechanism of PPARγ.

## Conclusion

In summary, although rosiglitazone stimulated StAR expression in human GLCs, the results demonstrated that rosiglitazone did not significantly inhibit E_2 _and progesterone productions in cultured GLCs, Moreover, rosiglitazone significantly decreased the production of TNFα in human GLCs, suggesting that PPARγ may play a role in the regulation of GLCs functions through inhibiting proinflammatory factors. Further studies need to investigate the molecular regulating mechanism of PPARγ on the GLCs. Careful attention also needs to be paid to the possibility that long-term treatment with rosiglitazone in patients with NIDDM and PCOS might also cause unexpected side effects, such as ovarian dysfunction.

## Competing interests

The authors declare that they have no competing interests.

## Authors' contributions

CQ performed most of the experiments, contributed to analysis and interpretation of the data and drafting of the manuscript. SX contributed to the study planning and interpretation of the data and revision of the manuscript. CJ contributed to the study planning and revised it critically for intellectual content. WJ and CL participated in the study design and revision of the manuscript. WY provided human GCs and performed a part of the experiments. SZ designed the study and contributed to interpretation of the data and writing of the manuscript. All authors read and approved the final manuscript.
